# Cardiac MRI Across ESC Guidance in the Last Decade

**DOI:** 10.1002/clc.70329

**Published:** 2026-05-18

**Authors:** Alexander Gall, Nicholas Sawh, Rui Li, Peter P. Swoboda, Andrew J. Swift, Gareth Matthews, Pankaj Garg

**Affiliations:** ^1^ Department of Cardiology Norfolk and Norwich University Hospitals NHS Foundation Trust Norwich Norfolk UK; ^2^ Department of Cardiovascular and Metabolic Health, Norwich Medical School University of East Anglia Norwich Norfolk UK; ^3^ Leeds Institute of Cardiovascular and Metabolic Medicine University of Leeds Leeds UK; ^4^ Division of Clinical Medicine, Immunity & Cardiovascular Disease University of Sheffield Sheffield UK; ^5^ National Institute for Health and Care Research Sheffield Biomedical Research Centre Sheffield UK

**Keywords:** cardiac imaging, cardiomyopathies, cardiovascular magnetic resonance, ESC guidelines, heart failure, tissue characterization

## Abstract

**Aims:**

Cardiovascular magnetic resonance (CMR) has shifted from a problem‐solving modality to a foundational, first‐line test in European Society of Cardiology (ESC) guidance. This review synthesises how CMR is positioned within ESC guidelines (2015–2025) and allied EACVI/EHRA statements, emphasizing its decision‐shaping role through accurate biventricular volumetry, tissue characterization (late gadolinium enhancement [LGE], T1/T2 mapping, extracellular volume [ECV]), and flow quantification (phase‐contract, with emerging 4D‐flow).

**Methods:**

We performed a structured narrative review of adult ESC clinical practice guidelines and allied expert statements (January 2015–December 2025) containing explicit CMR recommendations. Documents were identified via society portals and PubMed, excluding pediatric guidance and non‐recommendation technical notes. Two independent reviewers extracted data on clinical context, CMR role, and recommendation Class/Level of Evidence, resolving discrepancies by consensus.

**Results:**

The search identified 13 ESC guidelines—spanning coronary syndromes, sports cardiology, cardio‐oncology, myocarditis, and valvular disease—and 13 supporting statements. The data demonstrate a progressive elevation of CMR to a strongly favored investigation (commonly Class I/IIa, Level B/C) in scenarios where precision imaging alters management. Allied expert statements standardize protocols, reference ranges, and quantification methods to facilitate consistent clinical implementation.

**Conclusion:**

ESC guidelines have repositioned CMR from a tool of last resort to a cornerstone of cardiovascular diagnostics. Its adjudicatory role leverages tissue characterization and quantification to resolve clinical scenarios with high diagnostic uncertainty, including MINOCA, suspected cardiomyopathy, sports participation after myocarditis, and complex valvular disease. Expert statements provide the practical framework for implementation, supporting the progressive integration of CMR into clinical pathways.

## Graphical abstract

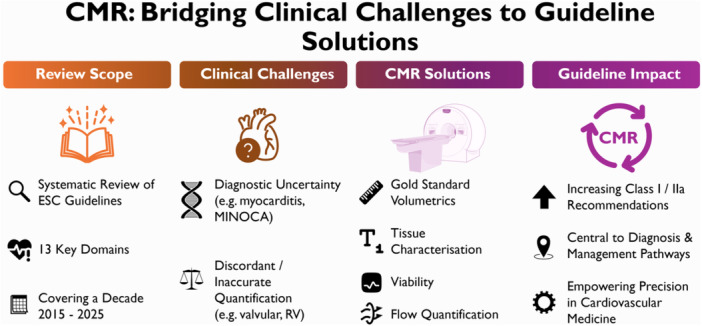

**Abbreviations**
AR: aortic regurgitation
CMR: cardiovascular magnetic resonance
ECV: extracellular volume fraction
HFpEF: heart failure with preserved ejection fraction
LGE: late gadolinium enhancement
MINOCA: myocardial infarction with non‐obstructive coronary arteries
MR: mitral regurgitation
NSTE‐ACS: non‐ST elevation acute coronary syndrome
PET: positron emission tomography
STEMI: ST‐elevation myocardial infarction
VT: ventricular tachycardia

## 1 Introduction

Cardiac imaging is a cornerstone of modern cardiology, playing a crucial role in the diagnosis, management, and prognostication of cardiovascular disease. Multimodality imaging, which integrates the complementary strengths of echocardiography, computed tomography, nuclear imaging, and cardiovascular magnetic resonance (CMR), provides a comprehensive assessment of cardiac structure and function. Among these techniques, CMR has evolved dramatically over the past decade. Once considered a tertiary, problem‐solving modality, it has become a vital tool for diagnosing, phenotyping, and risk‐stratifying a wide range of cardiovascular disorders [1, 2, 3].

A single, comprehensive CMR examination delivers a multiparametric assessment. Cine imaging provides reference‐standard quantification of ventricular volumes and systolic function. Myocardial tissue characterization by CMR is now intrinsically multiparametric, using complementary techniques to interrogate different pathological processes [1, 3]. T2‐weighted STIR imaging and quantitative T2 mapping facilitate the assessment of myocardial edema. LGE identifies focal scar and replacement fibrosis, a cornerstone for diagnosing ischaemic myocardial infarction and sub‐phenotyping non‐ischaemic cardiomyopathies. Quantitative parametric methods, including native T1 mapping and extracellular volume (ECV), enable assessment of diffuse fibrosis, infiltrative disease, and myocardial burden in disorders such as amyloidosis [2, 4]. Finally, perfusion and phase‐contrast techniques quantify ischemia, valvular regurgitation, intracardiac shunts, and complex vascular hemodynamics, with growing clinical interest in quantitative stress perfusion and 4D‐flow approaches [5].

### 1.1 Expansion of Clinical Use

As CMR is a non‐invasive, nonionizing test that offers enhanced precision across several cardiovascular disorders, it is not surprising that its clinical use is rising substantially worldwide. Its global clinical adoption has expanded substantially over the past decade. A recent international survey of clinical practice confirmed this trend, reporting increasing availability of CMR services—particularly driven by adult cardiology referrals—while also highlighting ongoing disparities in access, infrastructure, and specialist training [6]. Evidence from the Society for Cardiovascular Magnetic Resonance global registry has further demonstrated the tangible clinical impact of CMR in heart failure, with nearly half of all scans leading to a change in diagnosis or subsequent management [7]. Similarly, data from the earlier EuroCMR registry highlighted not only a high diagnostic yield and frequent therapeutic reclassification, but also an excellent safety profile in routine clinical settings [8]. Collectively, these findings have underpinned the progressive elevation of CMR's role in European Society of Cardiology (ESC) guidelines—from a modality to be “considered” in select scenarios to a frequently recommended investigation in a growing number of clinical pathways. Against this background, the present review is, to our knowledge, the first to map ESC guideline positioning of CMR across adult cardiology up to 31 December 2025 while explicitly pairing core guideline recommendations with European Association of Cardiovascular Imaging (EACVI)/European Heart Rhythm Association (EHRA)/EuroCMR implementation documents. We also emphasize decision‐shaping scenarios—such as myocardial infarction with non‐obstructive coronary arteries (MINOCA), arrhythmic mitral valve prolapse/mitral annular disjunction, non‐dilated left ventricular cardiomyopathy, non‐invasive left ventricular filling pressure assessment, sports participation after myocarditis, and cardio‐oncology phenotyping—in which CMR moves beyond diagnosis to alter management [9, 10, 11, 12, 13, 14].

## 2 Methods

### 2.1 Study Design

We set out to define how CMR is positioned across ESC guidance over the past decade and how allied European statements translate those recommendations into practice. This was a structured narrative review of adult cardiology guidelines and consensus documents published between January 1, 2015, and December 31, 2025.

### 2.2 Ethics Approval and Consent to Participate

Not applicable for this review of published guidelines and consensus statements.

### 2.3 Settings and Participants

Eligible sources were ESC clinical practice guidelines that contained explicit CMR‐related recommendations for diagnosis, risk stratification, treatment planning, procedural planning, or follow‐up. We also included EACVI, EHRA, and EuroCMR statements where CMR was a central component, as well as joint society documents listing these bodies as collaborators. Pediatric‐only guidance, editorials, technical notes without clinical recommendations, and superseded ESC guideline versions were excluded from the primary extraction set. When multiple versions existed, only the most recent document published within the review window was retained for extraction; earlier versions were consulted solely to confirm temporal changes in recommendation class, level, or scope.

### 2.4 Outcomes

Documents were identified through structured searches of the ESC, EACVI, EHRA, and EuroCMR guideline portals, followed by verification on PubMed. Search strings combined society names with disease domains and imaging terms and were iterated for cardiomyopathies, heart failure, valvular heart disease, coronary syndromes, arrhythmia, adult congenital heart disease, sports cardiology, cardio‐oncology, pulmonary hypertension, myocarditis/pericarditis, and endocarditis to ensure saturation. Given that this was a structured narrative review of guideline and consensus documents rather than a systematic review of primary studies, a formal PRISMA checklist was not applied; however, search, eligibility, extraction, and reconciliation steps were prespecified to enhance transparency and reproducibility. When an item was initially captured via a PubMed Central (PMC) record, we resolved and recorded the corresponding PubMed identifier (PMID) to maintain a uniform citation format.

For each eligible document, we extracted the clinical context, the stated role of CMR (diagnostic pathway placement, prognostic use, procedural planning, or follow‐up), the ESC recommendation Class (I/IIa/IIb/III), and Level of Evidence (A/B/C) exactly as written, and the PMID.

Terminology was harmonized by expanding abbreviations at first use and then adopting consistent forms throughout the dataset—for example: cardiovascular magnetic resonance (CMR), late gadolinium enhancement (LGE), ECV, and atrial fibrillation (AF). Disease areas were aligned with ESC taxonomy to enable comparison across documents that vary in structure and emphasis.

### 2.5 Statistical Analysis

Two reviewers independently completed separate structured extraction templates and were not formally blinded to each other's work or to source identity. Discrepancies in clinical phrasing, Class/Level transcription, or citation were reconciled by consensus; unresolved items were adjudicated by a third reviewer. To minimize transcription error, extracted Class/Level statements and PMIDs were rechecked against the source PDF or HTML version.

Given the heterogeneity of indications and endpoints, we used a narrative synthesis. For each disease area we report where CMR is positioned as first‐line versus problem‐solving, whether its primary function is diagnostic or prognostic, and where imaging results are explicitly linked to management decisions (e.g., timing of intervention in valvular disease or implantable cardioverter‐defibrillator consideration in hypertrophic cardiomyopathy). We then map EACVI/EHRA/EuroCMR statements onto these domains to show how technical standards, quantification methods, and consensus pathways implement guideline intent in practice.

## 3 Results

### 3.1 Primary Outcomes

**Figure 1:**
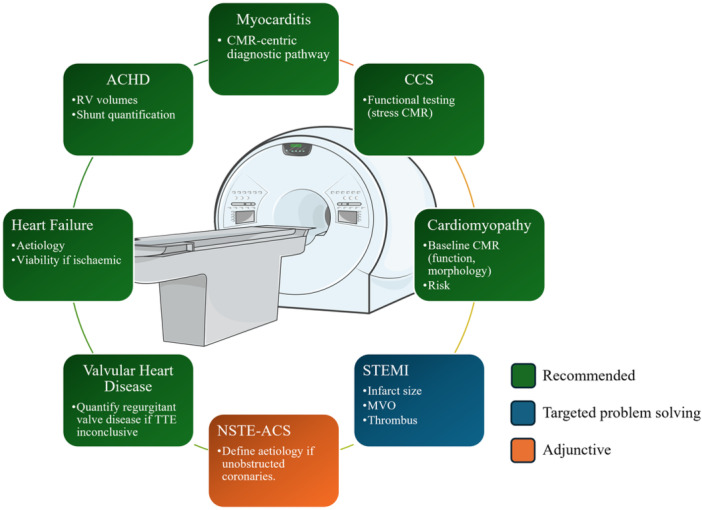
Overview of guideline indication for use of CMR based on common cardiac presentations, color‐coded based on class of recommendation (green = class I, blue = class IIa, orange = class IIb). Abbreviations: ACHD, adult congenital heart disease; CMR, cardiovascular magnetic resonance; CCS, chronic coronary syndrome; MVO, microvascular obstruction; NSTE‐ACS, non ST‐elevation – acute coronary syndrome; STEMI, ST‐elevation myocardial infarction; TTE, transthoracic echocardiogram.

**Figure 2:**
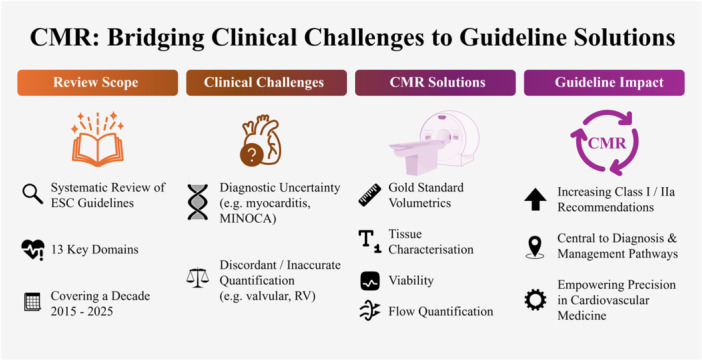
CMR: bridging clinical challenges to guideline solutions. Four panels summarise the role of cardiovascular magnetic resonance (CMR) in ESC guidance. Abbreviations: CMR, cardiovascular magnetic resonance; ESC, European Society of Cardiology; MINOCA, myocardial infarction with non‐obstructive coronary arteries; RV, right ventricle.

The structured search identified 13 distinct ESC clinical practice guidelines published between 2015 and 2025 that explicitly incorporate CMR within adult cardiology diagnostic or management pathways (Table 1). These guidelines span chronic coronary syndromes [15], acute coronary syndromes [9], sports cardiology [13], heart failure [16], cardiomyopathies [11], valvular heart disease [17], adult congenital heart disease [18], ventricular arrhythmias and sudden cardiac death [19], atrial fibrillation [20], cardio‐oncology [14], pulmonary hypertension [21], infective endocarditis [22], and myocarditis/pericarditis [23] (Figure 1). This breadth of inclusion reflects CMR's utility as a foundational modality for reproducible quantification of ventricular volumes and function, myocardial tissue characterization, and, increasingly, decision‐shaping pathway placement (Figure 2). Overall, the guidelines demonstrate a progressive shift, elevating CMR from a problem‐solving tool to a frequently recommended investigation (commonly Class I/IIa, Level B/C) in scenarios where precision in biventricular assessment, tissue typing, or flow quantification guides diagnosis and alters management decisions.

**Table 1:** Cardiac MRI in ESC guidelines (2015–2025): condition, clinical context, class/level.

Disease area & guideline	Clinical context for CMR	Role of CMR	Class/Level
Chronic coronary syndromes 2024 [15]	Suspected obstructive CAD; ANOCA/INOCA work‐up; functional ischemia assessment	Stress CMR as a frontline functional imaging test; accurate LV function when echocardiography is limited	I B (functional imaging); IIb C (LV function if echo inadequate)
Acute coronary syndromes 2023 [9]	MINOCA work‐up; selective post‐MI tissue characterization and risk assessment	CMR to define etiology (infarction vs myocarditis/Takotsubo/other injury); infarct size, MVO, intramyocardial hemorrhage, LV thrombus, and complications	I B (MINOCA evaluation); adjunctive/context‐specific post‐MI
Sports cardiology 2020 [13]	Athlete's heart vs cardiomyopathy; return‐to‐play after myocarditis/other cardiac disease	Differentiates physiological remodeling from pathology; scar and tissue characterization inform exercise restriction and return‐to‐play decisions	IIa C (context‐dependent)
Heart failure 2021 [16]	New HF; suspected infiltrative/inflammatory/storage disease; viability	Recommended when echo limited; tissue characterization (amyloid/sarcoid/myocarditis/iron); LGE to separate ischaemic vs non‐ischaemic DCM; stress CMR for ischemia/viability pre‐revascularisation	I C (core diagnostic); IIa C (LGE in DCM); IIb B (ischemia/viability)
Cardiomyopathies 2023 [11]	DCM/HCM/Arrhythmogenic/Restrictive/Non‐dilated LV cardiomyopathy	Baseline CMR (function, morphology, LGE, mapping/ECV); aetiological classification; scar‐based risk stratification	I C (core); additional IIa contexts
Valvular heart disease 2025 [17]	Discordant/inconclusive echo (MR/AR); RV assessment in TR; aortopathy; assessment of extravalvular consequences	Quantify regurgitation; ventricular volumes; reference standard for RV size/EF in significant TR; tissue characterization	I–IIa C (context‐dependent)
Adult congenital heart disease 2020 [18]	TOF/systemic RV; shunts; valves; great vessels	Method of choice for RV volumes/EF; Qp/Qs; regurgitation; aortic/great‐vessel imaging	I C
Ventricular arrhythmias/SCD 2022 [19]	VT/VF; substrate/ablation planning	Identify substrate (scar/inflammation); consider for ablation planning/risk	I–IIa B/C
Atrial fibrillation 2024 [20]	Pre‐ablation planning; selected post‐ablation assessment	CT or MRI for left atrial/pulmonary vein anatomy; MRI may add tissue phenotyping where feasible	I (pre‐ablation anatomy)
Cardio‐oncology 2022 [14]	Cancer‐therapy‐related cardiomyopathy/myocarditis; suspected cardiac masses; non‐diagnostic echocardiography	Tissue characterization, ventricular volumes, and mass assessment when echocardiography is limited or insufficient	IIa C (context‐dependent)
Pulmonary hypertension 2022 [21]	RV function/prognosis in expert centers	May be considered for RV volumes/EF, prognostication	IIb C
Infective endocarditis 2023 [22]	Adjunct in selected scenarios	Limited adjunctive use vs echo/CT/PET	—
Myocarditis and pericarditis 2025 [23]	Suspected myocarditis/pericarditis	CMR‐centric diagnostic pathway (Lake Louise Criteria 2018 with mapping); structured follow‐up	I C (context‐specific)

Abbreviations: ANOCA/INOCA, angina/ischemia with no or non‐obstructive coronary arteries; AR, aortic regurgitation; CMR, cardiovascular magnetic resonance; CAD, coronary artery disease; CT, computed tomography; DCM, dilated cardiomyopathy; ECV, extracellular volume; EF, ejection fraction; HCM, hypertrophic cardiomyopathy; HF, heart failure; LV, left ventricle; LGE, late gadolinium enhancement; MINOCA, myocardial infarction with non‐obstructive coronary arteries; MVO, microvascular obstruction; MR, mitral regurgitation; PET, positron emission tomography; RV, right ventricle; SCD, sudden cardiac death; TR, tricuspid regurgitation; TOF, tetralogy of fallot; VT, ventricular tachycardia; VF, ventricular fibrillation.

In parallel with the core ESC clinical practice guidelines, 13 complementary European expert consensus and position documents were identified, originating from or collaboratively involving specialized bodies such as EACVI, EHRA, and EuroCMR groups (Table 2). These supporting documents define the technical and methodological framework necessary for the practical implementation of the CMR recommendations established in the main guidelines. They cover critical areas, including standardization of imaging protocols and reference ranges—for example, EACVI CMR Reference Values and Grading [24] —definition of non‐invasive diagnostic criteria—for example, the updated Lake Louise Criteria for myocarditis [25] —and integration of CMR into complex multimodality pathways. Specific clinical foci addressed by these expert papers included electrophysiology and ablation planning—for example, the VT ablation consensus [26] —imaging guidance for AF [27] and electrophysiology procedures [28], phenotyping of cardiomyopathies and rare diseases—for example, arrhythmic MVP/MAD [10] and Anderson–Fabry disease [29] —multimodality imaging in women [30], multiple valvular heart disease [31], aortic stenosis [32], native valvular regurgitation [33], and assessment of myocardial viability [34]. Collectively, these consensus papers reinforce the clinical relevance of CMR by standardizing quantification methods for parameters such as LGE and flow, thereby facilitating their intended use in diagnosis, prognosis, and management planning.

**Table 2:** EACVI/EHRA/EuroCMR consensus/position documents with central CMR content (2016–2025).

Document [society; (citation)]	Focus	CMR‐specific contribution
Updated Lake Louise criteria (2018) [SCMR/EACVI [25]	Myocarditis	Defines mapping‐based criteria: ≥ 1 T2 (edema) and ≥ 1 T1 (injury: LGE/native T1/ECV) → establishes CMR as the non‐invasive reference framework.
Arrhythmic MVP/MAD (2022) [EHRA with EACVI collaboration [10]	Ventricular arrhythmias in MVP/MAD	Recommends CMR for fibrosis (LGE) in high‐risk MVP (prior arrest/sustained VT, syncope, NSVT) to inform ICD/ablation strategy.
VT ablation consensus (2019) [HRS/EHRA/APHRS/LAHRS [26]	Substrate mapping for VT	Advocates LGE‐CMR to delineate scar/heterogeneity and inform substrate‐guided ablation when feasible.
CMR reference values and grading (2019) [EACVI [24]	Standardisation	Provides reference ranges and severity grading for CMR‐derived chamber and aortic measurements—underpinning thresholds used in clinical decision‐making.
Multimodality Imaging in AF (2016) [EACVI/EHRA [27]	AF pathways	Details CT/MRI use for LA/PV anatomy pre‐ablation; discusses advanced MRI assessment (atrial tissue), placing CMR in a multimodality AF framework.
Pre‐/Post‐Procedural CT/MRI in EP (2024) [EACVI/EHRA [28]	EP procedural planning and follow‐up	Summarises the roles of CT/MRI to plan and evaluate EP procedures; standardizes pre‐procedural anatomy and post‐procedural assessment where MRI adds value.
Multimodality imaging in AFD (2025) [EACVI [29]	Rare disease imaging framework	Positions CMR for phenotyping, risk stratification, and therapy monitoring, including serial assessment of myocardial involvement.
Multimodality imaging in women (2024) [EACVI [30]	Sex‐specific considerations	Highlights scenarios where CMR adds unique value, including pregnancy, post‐partum disease, microvascular dysfunction, and diastolic phenotyping.
Native valvular regurgitation—MMI guidance (2022) [EACVI/ESC council VHD [33]	Standardized measurement set	Standardizes multimodality assessment and emphasizes where CMR quantification (regurgitant volume/fraction) influences decision‐making.
Aortic stenosis—EACVI clinical scientific update (2023) [EACVI [32]	Valvular stenosis assessment	CMR evaluation of myocardial remodeling/fibrosis (LGE/T1) informs risk stratification and timing of intervention.
Multiple valvular heart diseases—EACVI Consensus (2025) [EACVI [31]	MMI pathways when more than one valve is affected	CMR for flow partitioning, RV/LV remodeling, and integrated assessment when more than one valve lesion coexists.
Pericardial disease—EACVI position paper (2015) [EACVI [41]	Pericardial pathology	CMR for edema/LGE, hemodynamic assessment; differentiates constriction vs restrictive physiology
Myocardial viability—EACVI expert consensus (2021) [EACVI [34]	Tissue viability assessment	Integrates PET/CT/CMR; positions LGE/T1/T2 mapping and perfusion for viability decision‐making

Abbreviations: AFD, Anderson‐Fabry disease; AF, atrial fibrillation; CMR, cardiovascular magnetic resonance; CT, computed tomography; ECV, extracellular volume; EP, electrophysiology; ICD, implantable cardioverter defibrillator; LGE, late gadolinium enhancement; LA, left atrium; LV, left ventricle; MVP, mitral valve prolapse; MAD, mitral annular dysjunction; NSVT, non‐sustained VT; PV, pulmonary vein; PET, positron emission tomography; RV, right ventricle; VT, ventricular tachycardia.

### 3.2 Secondary/Exploratory Outcomes

**Figure 3:**
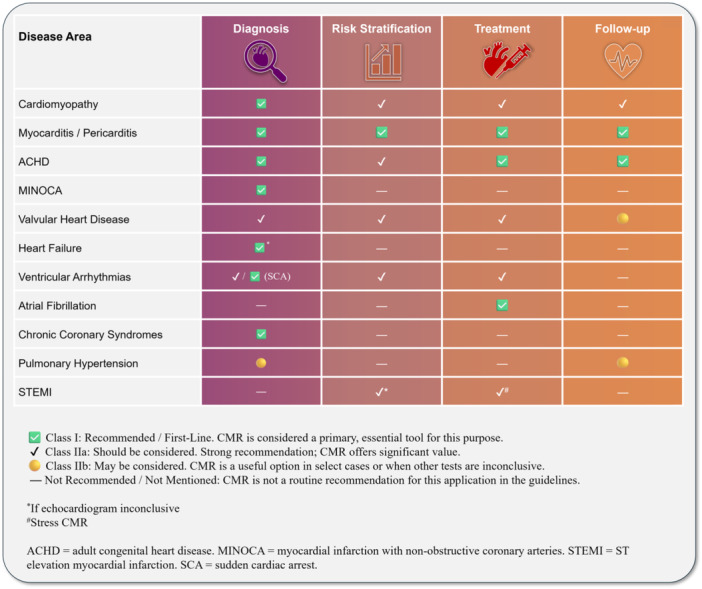
Summary of CMR positioning across major cardiovascular disease domains.

Two summary tables catalog (i) ESC guidelines and (ii) EACVI/EHRA/EuroCMR statements relevant to CMR, listing clinical context and class/level of recommendation (Tables 1 and 2; Figure 3). Evidence of real‐world impact from multicentre surveys and registries is summarised to contextualize adoption. These tables, along with Figure 3, provide a concise visual reference for readers and complement the primary narrative by illustrating patterns of guidance and implementation across disease areas.

## 4 Discussion

### 4.1 Principal Findings: ESC Guidelines

In this review, we consolidated the contemporary ESC guidelines that highlight the clinical utility of CMR across cardiovascular disorders and mapped how CMR is operationalized within diagnostic and therapeutic pathways through 31 December 2025 (Figure 3). We observed a consistent pattern: CMR is preferentially recommended when imaging must adjudicate competing aetiologies with management consequences, deliver quantitative thresholds that guide intervention, or uncover substrates with prognostic and procedural relevance. Across disease areas, its adoption reflects durable strengths—reproducible volumetry without geometric assumptions, multiparametric tissue characterization, and quantitative flow/hemodynamics—together with expanding real‐world evidence of management impact [6, 7, 8]. Notwithstanding this progress, several recommendations remain at Level C, underscoring the need for prospective, management‐linked trials that translate CMR metrics into outcome‐anchored decision thresholds.

In chronic coronary syndromes, the 2024 ESC guidance positions stress CMR alongside other frontline functional tests for patients with suspected obstructive coronary artery disease and gives greater visibility to patients with angina/ischemia and no or non‐obstructive coronary arteries (ANOCA/INOCA), a setting in which perfusion CMR is increasingly valuable [5, 15]. The 2023 acute coronary syndrome guideline unifies prior STEMI and NSTE‐ACS pathways and reinforces the role of CMR in structured evaluation of MINOCA, where it helps distinguish infarction from myocarditis, Takotsubo syndrome, or other non‐ischaemic injury, while also providing selective post‐infarct prognostic information such as infarct size, microvascular obstruction, intramyocardial hemorrhage, and left ventricular thrombus [9, 35].

The 2023 cardiomyopathy guidelines highlight CMR's central diagnostic role across phenotypes. Baseline CMR is recommended in suspected cardiomyopathy to define morphology, function, and scar burden; mapping‐based techniques (native T1/T2 and ECV fraction) enhance detection of diffuse processes that may evade LGE alone, refining aetiological attribution, family screening, and risk stratification [2, 11]. This is especially important in non‐dilated left ventricular cardiomyopathy, where CMR can reveal non‐ischaemic scar, fatty change, or subtle structural abnormalities despite the absence of overt chamber dilatation, thereby converting an apparently indeterminate phenotype into a guideline‐defined disease entity with implications for arrhythmic risk and device decisions [11, 36, 37]. In heart failure pathways, CMR is endorsed where echocardiography is limited and is specifically recommended for suspected infiltrative, inflammatory, or storage disease; when viability assessment could alter revascularisation decisions, CMR offers an evidence‐based alternative to other modalities [16, 34].

For valvular heart disease, the 2025 ESC/EACTS guideline recommends CMR when echocardiographic assessment is inconclusive or discordant with symptoms—especially in mitral and aortic regurgitation—because phase‐contrast flow quantification (regurgitant volume/fraction) and reference‐standard ventricular volumes may define the need to intervene [17, 33]. The same quantitative strengths underpin its role in adult congenital heart disease, where accurate assessment of right‐ventricular volumes/ejection fraction, pulmonary regurgitation, shunt fraction (Qp: Qs), and great‐vessel anatomy are pivotal for timing interventions, including pulmonary valve replacement in repaired tetralogy of Fallot or surveillance of a systemic right ventricle [18].

Arrhythmia guidance integrates CMR to reveal structural substrates that drive risk and procedural planning. The 2022 guideline on ventricular arrhythmias and sudden cardiac death assigns LGE‐CMR a key role in refining risk in dilated cardiomyopathy, hypokinetic non‐dilated cardiomyopathy, hypertrophic cardiomyopathy, and arrhythmogenic cardiomyopathy, and in delineating substrate to guide ablation [19, 36, 37]. In atrial fibrillation, the 2024 ESC guideline continues to support cross‐sectional imaging (CT or MRI) of the left atrium and pulmonary veins before ablation, with MRI offering the additional attraction of tissue‐based phenotyping where local expertise permits [20, 27, 28]. The 2020 sports cardiology guideline extends CMR into a distinct decision‐making arena: differentiating physiological athlete's heart from cardiomyopathy and informing return‐to‐play after myocarditis or other conditions in which tissue characterization and scar burden determine risk [13, 25].

CMR has become the non‐invasive reference framework for inflammatory myocardial disease. The 2018 scientific update to the Lake Louise Criteria codified a dual‐criterion approach—T2‐based edema plus T1‐based injury/fibrosis indices (native T1, ECV, or LGE)—to maximize diagnostic accuracy, and this framework is now embedded across ESC pathways [25]. The 2025 ESC guideline on myocarditis and pericarditis consolidates these roles across diagnosis, longitudinal follow‐up, and return‐to‐activity decisions, while recognizing intersections with invasive testing when CMR is not sufficient or feasible [13, 23]. In pericardial syndromes, quantitative tissue characterization is also maturing beyond conventional enhancement imaging, with recent data suggesting that mapping‐based approaches may further refine phenotyping in acute and recurrent pericarditis [38].

The 2022 cardio‐oncology guideline formalizes CMR as an important adjunct when echocardiography is non‐diagnostic or when tissue characterization, ventricular volumes, or cardiac mass definition can alter oncological or cardiovascular management, particularly in suspected cancer‐therapy‐related cardiomyopathy, myocarditis, or cardiac tumors [14]. In pulmonary hypertension, CMR‐derived right‐ventricular size/function and stroke‐volume metrics carry prognostic value and can be trended to monitor treatment response in expert centers, reflecting their incorporation in the 2022 ESC/ERS guideline [21]. The 2023 endocarditis guideline formalizes a multimodality imaging strategy in which MRI primarily augments detection of extracardiac complications (e.g., cerebrovascular embolic events), complementing echocardiography, CT, and positron emission tomography according to clinical scenario [22].

### 4.2 EACVI/EHRA/EuroCMR Position Statements

CMR is positioned across the EACVI/EHRA literature as the modality that resolves clinical uncertainty when morphology alone is not enough and when tissue characterization, hemodynamic assessment, or volumetric precision can change decisions. The arrhythmia pathway illustrates this most clearly. The EACVI/EHRA atrial fibrillation consensus formalized the use of left atrial phenotyping with cine CMR and atrial LGE—not to replace electroanatomic mapping, but to set expectations for ablation by relating pre‐ablation fibrosis burden to recurrence risk and by providing a framework to understand failures when scar is extensive [27]. The subsequent EHRA consensus on pre‐ and post‐procedural CT/CMR codifies practical, sequence‐level guidance on when to use cine and LGE‐CMR to define ventricular scar architecture (location, transmurality, and border‐zone), how to integrate segmented scar into electroanatomic maps, and where CT's spatial resolution and epicardial fat depiction can be advantageous; it also draws sensible guardrails around atrial LGE reproducibility and the still‐evolving evidence that image integration improves outcomes [28]. In ventricular arrhythmia ablation, the VT ablation consensus places LGE‐CMR at the centre of substrate delineation—particularly for intramural and non‐ischaemic scar—normalising pre‐procedural CMR to refine aetiology in non‐ischaemic cardiomyopathy, plan access routes, and identify conduction channels that determine ablation strategy [26].

In valvular heart disease, CMR plays complementary yet decisive roles. The EACVI native valvular regurgitation document emphasizes CMR as the adjudicator when echocardiography is discordant or borderline, because phase‐contrast flow and reference‐standard volumetry reduce misclassification; CMR‐derived regurgitant volumes and fractions are framed as management‐directing numbers rather than ancillary measurements [33]. For arrhythmic mitral valve prolapse/mitral annular disjunction (MVP/MAD), the joint consensus centers risk on CMR's ability to verify MAD geometry, quantify remodeling, and—critically—detect LGE in the papillary muscles and peri‐annular regions, the pattern with the clearest mechanistic link to malignant ventricular arrhythmias. The document is explicit about protocols (multi‐plane long‐axis cines; comprehensive tissue characterization) and about clinical triggers for CMR (survivors of sudden cardiac death (SCD), sustained ventricular arrhythmia, unexplained syncope/non‐sustained VT, or high‐risk phenotypic features), reflecting a shift from “nice‐to‐have” imaging to targeted risk work‐up [10]. In aortic stenosis, EACVI purposefully extends the focus from valvular severity to the myocardial response, positioning mid‐wall LGE, native T1/ECV, and—in advanced centers—4D‐flow as tools that inform timing of intervention with respect to myocardial health, not gradient alone [32].

For ischaemic heart disease and hibernation, the EACVI viability statement reiterates CMR's role as the reference framework for tissue viability: LGE transmurality to set an upper bound on functional recovery and mapping‐based techniques to refine the grey zone where scar is partial or diffuse. This pragmatic schema is particularly useful when PET is unavailable or where multimodality arbitration is required to balance procedural risk with realistic expectations of contractile improvement [34].

Inherited and metabolic cardiomyopathies are another domain where CMR progresses from diagnosis to disease modification. Beyond its established role in amyloidosis, where parametric mapping carries growing prognostic value [4], CMR is central in rare‐disease pathways. In Anderson–Fabry disease (AFD), EACVI codifies a CMR‐first approach to myocardial involvement: low native T1 as an early hallmark of sphingolipid storage, basal inferolateral LGE as a marker of irreversible injury, T2‐based signals of active inflammation, and serial CMR to monitor therapy response and inform risk [29]. Importantly, the document aligns with an emerging body of work showing that CMR can non‐invasively define left ventricular filling pressure (LVFP/pulmonary capillary wedge pressure [PCWP]), bridging structure and physiology and offering a determinant that tracks symptoms and outcomes beyond anatomy alone [29]. The EACVI position paper on cardiovascular imaging in women makes a similar pivot: it emphasizes the advantages of a nonionizing modality across the reproductive years, underscores sex‐specific phenotypes where microvascular disease and diastolic dysfunction are prevalent, and explicitly references invasively validated CMR‐based LVFP models that connect diastolic burden to prognosis and symptom limitation in women [30].

The original LVFP literature gives this physiological turn its evidentiary backbone. An invasively validated, physiology‐driven CMR model demonstrated that non‐invasive estimation of LVFP aligns with pulmonary capillary wedge pressure and that elevated CMR‐LVFP portends worse outcomes, reframing CMR as a tool that can recover load conditions rather than only depict structure [12]. Subsequent multicentre validation extended these findings to symptom associations and prognostic discrimination in heart failure with preserved ejection fraction (HFpEF), strengthening the clinical‐utility signal and matching the priorities articulated in the Women and AFD documents [39].

Taken together, these EACVI/EHRA statements are consistent in both opportunity and restraint. They elevate CMR from adjunct to arbiter when decisions hinge on substrate biology (fibrosis patterns in MVP/MAD; transmural and intramural scar in VT), when quantification must be reference‐standard (regurgitant burden in MR/AR), or when hemodynamics are otherwise invisible (LVFP in HFpEF/AFD; microvascular ischemia syndromes in women). At the same time, they define the boundaries of best practice: acknowledge reproducibility challenges for atrial LGE, leverage CT's strengths where appropriate, and call for prospective outcome studies where image integration is intuitively valuable but not yet trial‐proven [27, 28]. This integrated view aligns closely with the ESC‐guideline trajectory and helps explain why CMR now sits not only as a diagnostic modality but as a decision‐shaping instrument across electrophysiology, valve disease, ischemia, inherited/metabolic cardiomyopathies, and sex‐specific cardiovascular care.

### 4.3 Future Directions

Implementation and health‐system considerations remain salient. The wider availability of MRI‐conditional devices, streamlined protocols (including abbreviated, question‐focused scans), and maturing post‐processing standards have reduced barriers to adoption; yet, persistent constraints in scanner capacity and specialist workforce contribute to utilization heterogeneity [40]. Aligning local pathways with standardized measurement and reporting guidance and severity grading from European imaging societies can narrow these gaps while preserving the class/level intent of ESC recommendations. Looking forward, clinically validated 4D‐flow and AI‐assisted analytics could extend CMR's quantitative edge—particularly for complex valvular jets and aortopathy—provided they demonstrate reproducibility, interoperability, and incremental patient‐level value beyond contemporary best practice.

### 4.4 Limitations

Class/Level gradings are indication‐specific within each guideline. We report the dominant grading for common scenarios; readers should consult the full guideline when operating at edge cases (e.g., complex congenital lesions, device interactions, or investigational techniques such as 4D‐flow).

This review is limited to guidelines from the ESC and associated European bodies. It does not include recommendations from other major international societies, such as the American Heart Association/American College of Cardiology (AHA/ACC), which may differ in scope or emphasis.

Many CMR recommendations, particularly in emerging areas, are based on Level of Evidence C (expert consensus). This reflects the logistical challenges of conducting large‐scale, randomized trials for imaging modalities but underscores the need for more robust, outcome‐based evidence.

Guideline recommendations do not always reflect real‐world clinical practice, which is often influenced by local expertise, scanner availability, and healthcare system reimbursement. This review cannot capture the heterogeneity of CMR implementation across Europe.

Our analysis is limited to documents published up to December 2025. The field of CMR is rapidly evolving, and new evidence or techniques may have emerged since the publication of the latest guidelines included in this review.

## 5 Conclusion

Over the last decade, ESC guidelines have fundamentally repositioned CMR from a tool of last resort to a cornerstone of cardiovascular diagnostics. Its role is no longer adjunct but often adjudicatory, leveraging tissue characterization and quantification to resolve the clinical scenarios—such as MINOCA, suspected cardiomyopathy, return‐to‐play after myocarditis, cardio‐oncology phenotyping, and discordant valvular disease—that carry the greatest diagnostic uncertainty and management consequence. The expert consensus documents from EACVI and EHRA run in parallel, providing the practical framework to implement these recommendations. While persistent challenges in access, standardization, and the need for more robust trial‐derived evidence remain, the progressive integration of CMR into clinical pathways is undeniable. The increasing prominence of CMR within ESC guidance reflects a broader shift towards imaging that not only visualizes anatomy but also quantifies physiology and defines tissue substrate.

## Author Contributions

Study conception: Alexander Gall, Gareth Matthews, and Pankaj Garg. Study design and protocol development: Alexander Gall and Pankaj Garg. Statistical analysis: Nicholas Sawh and Rui Li. Administrative support: Peter P. Swoboda and Andrew J. Swift. Supervision: Gareth Matthews and Pankaj Garg. Data collection: Alexander Gall, Nicholas Sawh, and Rui Li. Data analysis: Peter P. Swoboda, Andrew J. Swift, and Gareth Matthews. Drafting of manuscript: Alexander Gall, Nicholas Sawh, and Pankaj Garg. Critical review of the manuscript and reviewed and approved the final version of the manuscript: all authors. Pankaj Garg takes responsibility for the overall content as guarantor.

## Conflicts of Interest

Dr. P. Garg is a clinical advisor for Pie Medical Imaging and Medis Medical Imaging. Dr. P. Garg consults for Anteris Technologies and Edwards Lifesciences. Dr. A. Gall, Dr. G. Matthews, and Dr. R. Li consult for Edwards Lifesciences.

## Data Availability

Data sharing is not applicable to this article as no datasets were generated or analyzed during the current study. No new data was generated or analyzed for this review.
